# Metrology part 2: Procedures for the validation of major measurement quality criteria and measuring instrument properties

**DOI:** 10.1007/s10877-020-00495-x

**Published:** 2020-03-18

**Authors:** Pierre Squara, Thomas W. L. Scheeren, Hollmann D. Aya, Jan Bakker, Maurizio Cecconi, Sharon Einav, Manu L. N. G. Malbrain, Xavier Monnet, Daniel A. Reuter, Iwan C. C. van der Horst, Bernd Saugel

**Affiliations:** 1 Department of Cardiology and ICU, Clinique Ambroise Paré, Neuilly-sur-Seine, France; 2 Department of Anesthesiology, University of Groningen, University Medical Centre Groningen, Groningen, The Netherlands; 3 Intensive Care, St Georges’ University Hospitals NHS Foundation Trust, London, UK; 4 Departmento de Medicina Intensiva, Facultad de Medicina, Pontificia Universidad Católica de Chile, Santiago, Chile; 5 Department of Intensive Care Adults, Erasmus MC University Medical Center, Rotterdam, The Netherlands; 6 Department of Pulmonary and Critical Care, New York University, New York, USA; 7 Division of Pulmonary, Allergy, and Critical Care Medicine, Columbia University Medical Center, New York, USA; 8 Department of Anesthesia and Critical Care, Humanitas University, Milano, Italy; 9 General Intensive Care Unit of the Shaare Zedek Medical Centre, Hebrew University Faculty of Medicine, Jerusalem, Israel; 10 Department of Intensive Care, University Hospital Brussels (UZB), Jette, Belgium; 11 Faculty of Medicine and Pharmacy, Vrije Universiteit Brussel (VUB), Brussels, Belgium; 12 Medical Intensive Care Unit, Paris-Sud University Hospitals, Assistance Publique-Hôpitaux de Paris, Inserm UMR S_999, Le Kremlin-Bicêtre, France; 13 Department of Anesthesiology and Intensive Care Medicine, University Medical Center Rostock, Rostock, Germany; 14 Department of Intensive Care, Maastricht University Medical Center+, Maastricht, The Netherlands; 15 Department of Anesthesiology, Center of Anesthesiology and Intensive Care Medicine, University Medical Center Hamburg-Eppendorf, Hamburg, Germany; 16 Outcomes Research Consortium, Cleveland, OH USA

**Keywords:** Statistics, Critical care, Perioperative medicine, Hemodynamic monitoring, Cardiovascular dynamics

## Abstract

A measurement is always afflicted with some degree of uncertainty. A correct understanding of the different types of uncertainty, their naming, and their definition is of crucial importance for an appropriate use of the measuring instruments. However, in perioperative and intensive care medicine, the metrological requirements for measuring instruments are poorly defined and often used spuriously. The correct use of metrological terms is also of crucial importance in validation studies. The European Union published a new directive on medical devices, mentioning that in the case of devices with a measuring function, the notified body is involved in all aspects relating to the conformity of the device with the metrological requirements. It is therefore the task of scientific societies to establish the standards in their area of expertise. After adopting the same understandings and definitions (part 1), the different procedures for the validation of major quality criteria of measuring devices must be consensually established. In this metrologic review (part 2), we review the terms and definitions of validation, some basic processes leading to the display of an indication from a physiologic signal, and procedures for the validation of measuring instrument properties, with specific focus on perioperative and intensive care medicine including appropriate examples.

## Introduction

Verification is a provision of objective evidences that a given measuring instrument fulfills specified requirements [1]. Validation is a verification, where the specified requirements are adequate for the intended use [1]. Therefore, validating measuring instruments mandates to determine first the intended use. In perioperative and intensive care medicine, we are basically measuring different quantities coming from the patient with basically three main objectives

1. Monitoring: For monitoring purposes, we track the indication of a quantity value. When the quantity value changes and is outside a specified range it usually triggers an alarm. For example, we can decide that a systolic blood pressure value higher than 160 mmHg or lower than 90 mmHg triggers an (acoustic and/or visual) alarm.
2. Diagnosis and prognosis: For these purposes, we compare the indication of a quantity value to its expected range, with specified uncertainty. For example, we can assume that a normal SpO_2_ is 96–100%, measured with ± 2% uncertainty using a given measuring instrument, so that the diagnosis of hypoxemia is generally determined by an SpO_2_ < 94%.
3. Therapy: For therapeutic purposes we look at improving the indication of a quantity value up or down to a predetermined target value. For example, in a specific patient, we can decide to titrate FiO_2_ to reach and maintain a SpO_2_ > 94%.

These three objectives need acceptable instrumental properties but priorities can be made according to the objective. For monitoring purpose, indications are compared to their initial value, measured under similar conditions, and the delay for indicating significant directional changes generally matters. Therefore, instrumental precision, step response time (all linked to random errors of measurements), and stability are the first priorities. In contrast, for diagnostic and therapeutic purposes, since the measurand is compared with a predetermined absolute value, usually established from a reference method, the indication must be as close as possible to the true value (in the measuring interval). Therefore, instrumental bias, sensitivity (all linked to systematic errors of measurements), and their stability are the first priorities. For all purposes, selectivity is necessary.

Although not impossible, it is not usual to imagine promoting and validating a measuring instrument for a restricted use only (e.g., monitoring, diagnosis, or therapy). Consequently, the manufacturers of measuring instruments most often try to reach an acceptable compromise between the different instrumental properties (i.e., optimal compensation of systematic and random errors). However, until now, no consensual independent validation procedure addressing all these properties has been suggested. It is the task of a scientific society to promote the standards and to suggest such independent validation procedures.

## How is an indication obtained?

**Fig. 1:**
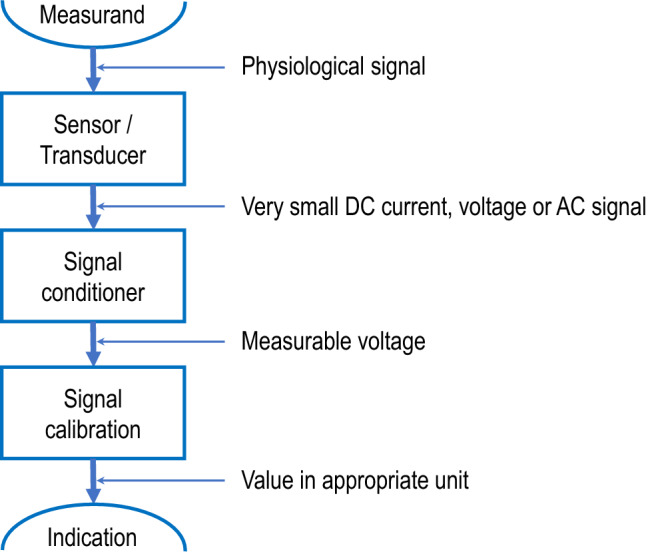
Schematic steps of the signal processing from the physiological signal to the measuring instrument indication

For a better understanding of the validation procedure requirements, it is necessary to share some basics on the main processes leading to the display of an indication from a physiologic signal (Fig. 1). Although they may have an impact on measurement errors, electronic processes not related to the physiologic signal such as power supply (excitation), electric isolation, compensation, or multiplexing are not within the scope of this document.

### Sensing

In medicine, most measurements start from a patient’s physiological signal analyzed by a sensor (rarely a detector which has only a binary response: present or not). Whatever the sensed physiological signal (electric waveform, temperature, flow velocity, pressure, tension, light absorption, sound, electrochemical reaction…), it usually transforms a constant electric current in a time-varying electric voltage (or conversely), allowing easier signal conditioning, which precludes quantitative analyses.

*Example* The arterial blood pressure signal (hydrostatic pressure signal) can be conducted by an arterial catheter and a fluid-filled tubing system from the artery to a sensor (for example quartz resonator changing frequency in response to stress) where it is transduced in an electric signal.

### Signal conditioning

The analog electrical output of a sensor-transducer is usually small in value and has non-idealities. The different steps of the signal conditioning aim at minimizing these non-idealities to make the signal representative of the physiological phenomenon and suitable for measurements. The objective is basically to increase as much as possible the signal-to-noise ratio. The different steps listed below are schematic and can be combined in various sequences.

#### Amplification

**Fig. 2:**
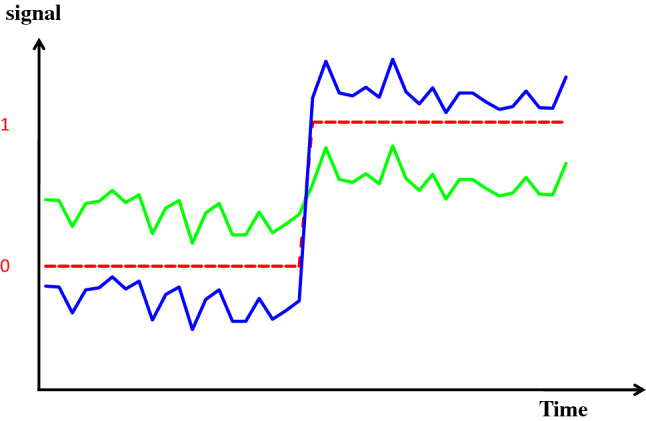
Schematic representation of amplification. In green the original transducer output is shown, with a signal-to-noise ratio = 10. The change of the signal represented in the middle of the figure is below the resolution of the analog-to-digital converter (with schematically 0,1 response represented by the red dotted line). In blue, the signal has been amplified (x5). The change of the signal can be perceived by the analog-to-digital converter. Since the noise (external noise) is not amplified, the signal-to-noise ratio is also improved by a factor of 5, allowing easier filtration

The analog output signal of a sensor-transducer is usually rather small in amplitude. Amplification will increase the amplitude/resolution of the signal to facilitate further analysis (Fig. 2). The tuning of output/input amplitude ratio is called span or gain adjustment.

#### Filtering

**Fig. 3:**
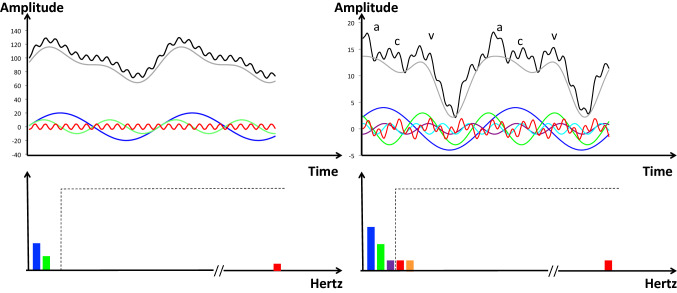
Schematic representation of Fourier filters. On the left, the black curve in the upper panel shows an example of periodic output current coming from a gauge measuring radial artery blood pressure. It can be mathematically decomposed into a very simple Fourier series of one fundamental sine wave (first harmonic) with a frequency of 1 Hz (blue curve), its second harmonic (2 Hz, green curve), and a high-frequency harmonic (frequency 50 Hz, in red), figuring an example of noise. The 1 Hz fundamental sine wave indicates that the heart rate is at 60 beats/min. If it was 90 or 120 beats/min, this fundamental sine wave would be 1.5 or 2 Hz, respectively. Similarly, it would be the fundamental sine wave, of all physiological variables related to heart contraction. In the lower panel, the frequency spectrum of the black curve can be created by plotting each sine wave amplitude in the y-axis and its frequency on the x-axis. Different electronic filters (low pass, high pass, band pass, band stop) may be applied to remove the energy of unwanted frequencies. The open box in dotted line shows the example of a low-pass filter allowing frequencies of 1–3 Hz and eliminating higher frequencies. The grey curve on the upper panel shows the blood pressure signal after filtration (presented with an offset for readability). On the right, the same representation and filtering for an example of left atrial pressure is shown. The decomposition results in three harmonics (1 Hz blue, 2 Hz green, 3 Hz purple), instead of 2. The noise (red curve) can also be decomposed in three components (only shown in the lower panel for clarity: 4 Hz red, 5 Hz orange, and 50 Hz red). a, c, v figure the main hemodynamic atrial waves: a, systolic contraction; c, isovolumic ventricular contraction; v, venous filling

Physiological signals are always corrupted by noises of various origin. The 50/60 Hz AC power lines are only the most common. Additive noise may come from other electric devices and patient-associated factors such as activity, respiration, heart rate, or high-frequency muscle contraction. In contrast to external noise (not due to the measuring system), internal noise results from the intrinsic technology. The aim of filtering is to remove as much as possible the noise and to keep as much as possible the pure physiological information to be measured. Choosing the appropriate filtering is dependent of the nature of both physiological signal and noise and it is a science on its own. A common example is the discrete-time Fourier transformation which enables to decompose any periodic signal into a set of sine waves, allowing to analyze the signal, not only in the time domain but also in the frequency domain. This may help in selecting the appropriate filtering methods (Fig. 3).

#### Linearization

Often, sensors do not have a linear relationship between the input physiological signal and the output voltage signal. The principle of linearization is to determine the relationship between the signal value and the quantity it is measuring, then to pass the signal through a circuit (or a mathematic formula) that has a response inverse to that of the measurement methodology. For example, if the transducer has an exponential response, its output signal might be passed through a mathematical formula/circuit that has a logarithmic response.

#### Analog-to-digital conversion

At any point of the signal processing that may be found optimal, a step of analog-to-digital signal conversion can be required to perform appropriate treatment using software and firmware, for communication, display, and storage of the information.

### Calibration

**Fig. 4:**
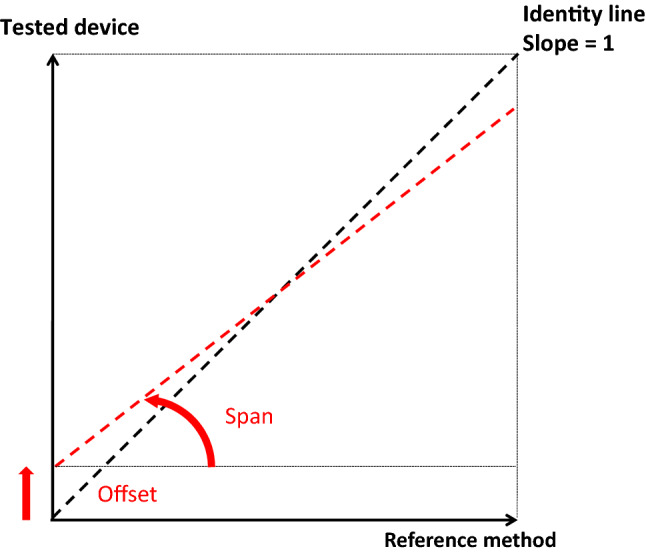
Schematic representation of offset and span for the adjustment of a measuring system

The signal needs then to be expressed proportionally and in the same units than the sensed quantity. In addition, the accuracy of all devices degrades over time due to the physiological response to the measuring system (clotting, fibrin deposit,…), instrumental drift, electric or mechanical shock, or a hazardous manufacturing environment. As described previously, calibration is a traceable comparison between a standard measurement and the device indication. The uncertainty of the measurement standard should be small, typically 10 times the accuracy of the test device. However, a ratio of 3:1 is acceptable for most standards organizations. Calibration is specific to each device. It may be expressed by a statement, calibration function, calibration diagram, calibration curve, or calibration table. Although strictly speaking it is a distinct process, in practice, calibration also includes the adjustment of the test device if necessary. A report is provided by the calibration expert, which shows the error in measurements with the measuring device before and after the calibration-adjustment. The adjustment has two basic steps schematized by drawing the calibration curve, plotting the measurement standard values on the x-axis and the test device on the y-axis. In the theoretical situation of a perfect reference method, the offset is the y-intercept, and the span is the regression slope, ideally tuned to 1 by amplification (Fig. 4). It is important to notice that calibration can only minimize systematic errors (improve trueness) and has no influence on random errors (precision).

### Averaging

Any indication given by a measuring device is most often the result of a combination of numerous sensed measurements during a specified period of time. For example, the heart rate in beats per minute can be determined by measuring the delay in milliseconds between two heartbeats and then by dividing 60,000 by this delay. Alternatively, the heart beats may be counted during a 10-s interval and the obtained number multiplied by 6. Depending on the physiologic signal to be measured, the time sampling may range from several milliseconds to several seconds. However, from these elementary measurements to the final indication displayed on the screen of the measuring device, different methods of averaging may be applied. To stick to the example of the heart rate, an indication changing on a monitor with every beat may be uncomfortable to read for medical caregivers. It can be preferable to make an averaging of the beat-by-beat analysis to refresh the indication less frequently. Moreover, to smooth this averaged value, the averaged sample may take into consideration the preceding results (moving average). There is a huge number of averaging methods, using mathematic or algorithmic tools, but averaging is always associated with a loss of information.

Finally, after appropriate calibration and adjustment, the metrological properties of a measuring device depend on the combined uncertainty of each elementary measurement, the sampling time, and the averaging method. The final compromise is always aimed at compensating the intrinsic limitations of the device. Basically, when the physiological signal sensed by the measuring system is poorly linked with the measurand, the precision of measurements is low, leading to an increase in sampling time and to the use of sophisticated averaging methods. This is always at the expense of resolution and step response time.

*Example* If elementary minute-measurements of blood flow decrease linearly from 6.0 to 4.0 L/min (6.0, 5.9, …, 4.0), a 20% decrease in blood flow is indicated in 7 min when there is no averaging (5.4 vs. 6) or in 8, 9, 12 min when 3-, 5-, and 10-min moving arithmetic averaging is used.

## Validation procedures

Regulatory requirements for labeling measuring instruments [CE marking for countries within the European Economic Area (i.e., States of the European Union plus Iceland, Norway, Liechtenstein, Switzerland and Turkey) or US Food & Drug Administration approval] are intended to assure the safety, effectiveness, and proper labeling of medical devices, in order to control exposure to potentially hazardous events, and to ensure the safe, efficacious use of such devices. The International Organization for Standardization (ISO) mandates that each manufacturer determines an intended purpose and ensures that the device is suitable for its intended purposes and capable of producing valid measurement results. However, for measuring instruments in perioperative and intensive care medicine, there is no independent definition of what is a “valid” result in clinical practice.

### Reference measurement procedure

For each specific measurand, a reference measurement procedure, usable in clinical conditions, must be identified. The reference measurement procedure must be recently and appropriately calibrated in an official national reference laboratory, used in the appropriate conditions and in its validated measuring interval. Its uncertainty must be known, traceable, and presumably small as compared to the uncertainty of the test device.

For example, for testing a new device for cardiac output assessment, an artificial heart or an extra-corporeal pump with a measurable flow may be chosen for giving a reference. If the test device requires a beating heart, an ultrasonic flow probe positioned around the pulmonary artery or the aorta during an open chest surgery may be preferred. If the test device requires a closed chest and standard clinical conditions, the dilution of a bolus indicator may be chosen. Most often, the reference value of the measurand is obtained by averaging several measurements from the reference procedure. The appropriate number of replicates must be chosen for reaching, at least, an uncertainty four times less than that of the test device (preferably ten times).

The number of replicate measurements with the reference method needed to derive the reference value of the measurand with the prescribed uncertainty is determined by the standard error of the mean (SEM) according to the formula: 2 SEM = 2 σ/$\sqrt{n}$ if the uncertainty is limited to imprecision. For example, if the test device has a presumable imprecision of 20% for determining cardiac output, the reference value must at least have an imprecision ≤ 5% (four times less), and preferably ≤ 2% (ten times less). Therefore, if the reference method has an imprecision of 10% (2σ), then the reference value (estimated true cardiac output) must average at least 4 reference measurements (objective = 5%, obtained by SEM = 10%/$\sqrt{4}$). Preferably, 25 reference measurements must be averaged to reach a SEM = 10%/$\sqrt{25}$ = 2%. Theoretically, a reference method can be found from continuous measurements if the high variability can be compensated by the large amount of data collected, therefore leading to an acceptable SEM [2].

### Constancy of the measurand

A measurement procedure takes a certain amount of time. The imprecision inherent to all measurements mandates averaging several measurements to determine the reference value and the tested quantity value of the measurand. During the collection of all the needed measurements, the measurand must be as constant as possible. If this is not the case, the dispersion of the measurand adds another dimension of uncertainty [3].

### The test device: estimation of measurements properties

In a unique patient in hypothetical clinical steady state, the needed number of replicate measurements must be performed using the reference method and the test device in parallel, during a short period of time. If we theoretically assume that during a stable, short period of time, the measurand is constant, the appropriate number of replicate values obtained from the reference method gives the reference value (or estimated true value, T) and its variability (2σ_ref_) due to random errors that may be called instrumental precision or uncertainty of the reference method.

#### Instrumental precision

**Fig. 5:**
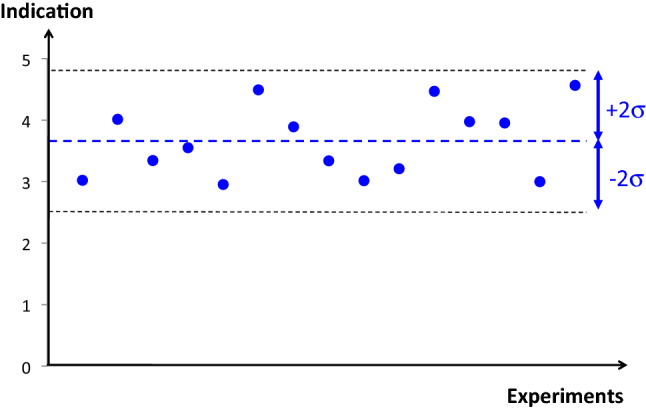
Schematic representation of instrumental precision by the variability of 15 cardiac output indications due to random errors. This dispersion is centered with the mean value without any need for a reference

Then, the mean quantity value obtained from the test device (µ) can be derived with its variability (2σ_Test_). This variability, in repeatedly assessing a constant quantity value of the measurand, is due to random errors of measurements. The mean value of random error is 0 if normally distributed around the mean value of the measurand µ, and refers to as the instrumental precision of the test device. This is an internal instrument property that needs no reference to be estimated (Fig. 5).

#### Instrumental bias

**Fig. 6:**
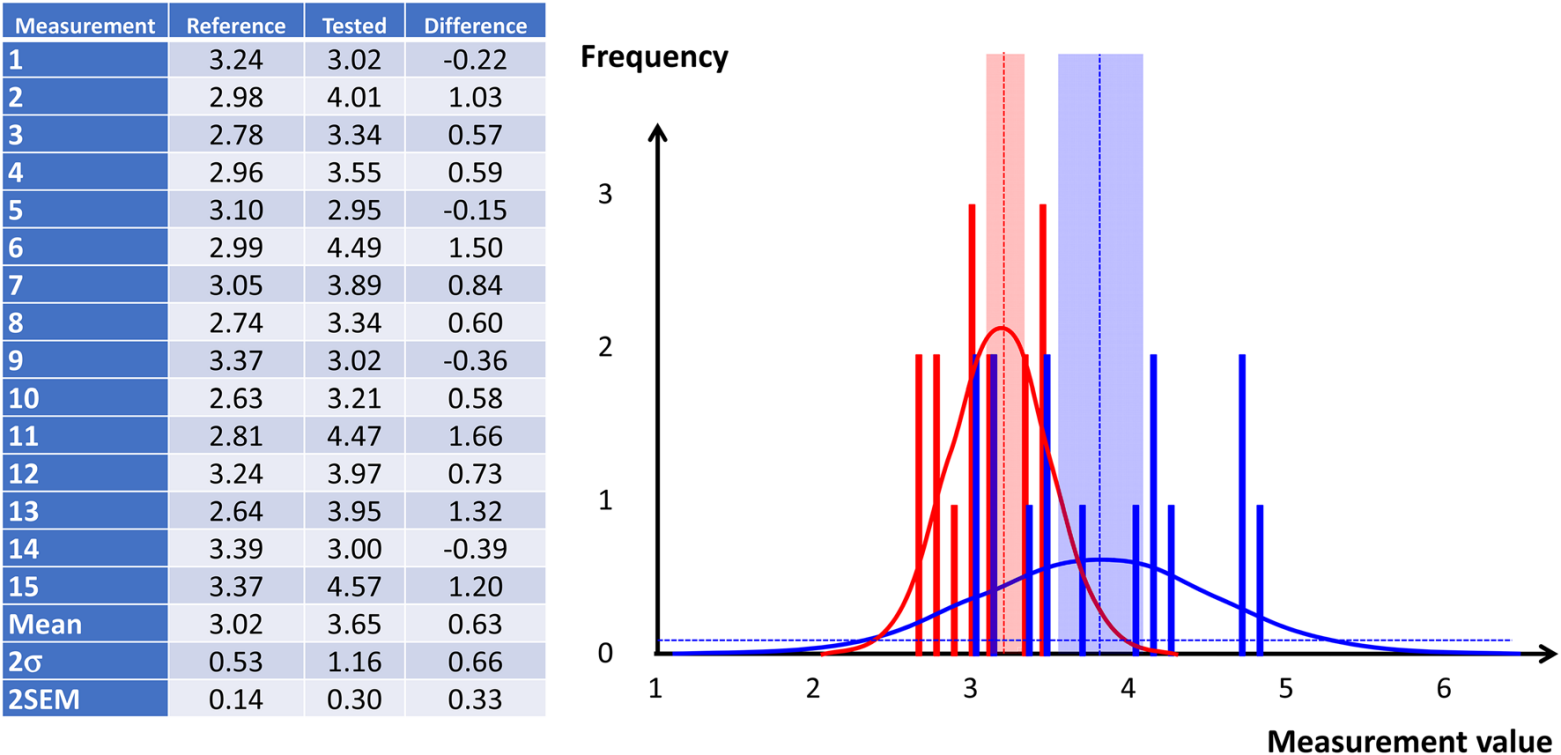
Comparison of a test device for measuring cardiac output against a reference method. In this example, it was chosen to collect the same number of measurements (15) using the reference method and using the test device (left panel table). On the right the corresponding frequency distributions with a step = 0.1 is shown (reference in red and test device in blue). Also figures are the normal adjustment (plain curve), the mean value (dotted line), and the 95% confidence interval (± 2SEM area around the mean value). In this example, the systematic error (instrumental bias) is 0.63, the uncertainty on this systematic error is given by the formula (SEM_ref_^2^ + SEM_Test_^2^)^0,5^ = 0.33, and the confidence interval [0.31–0.96], being significant with p < 0.001 using a Welch’s *t* test

The difference between µ and T evaluates the systematic error of the tested device indications and refers to as instrumental bias. The uncertainty on the systematic error depends on the respective SEMs, and thus on the confidence interval attributed to the reference method and to the confidence interval attributed to the test device (Fig. 6). The significance of the mean difference (instrumental bias) may be tested using a Welch’s *t *test. Indeed, the Student’s *t *test (and ANOVA) assumes that the two populations have normal distributions with equal variances. The Welch’s adaptation is designed for unequal variance still with the assumption of normality.

#### Sensitivity

**Fig. 7:**
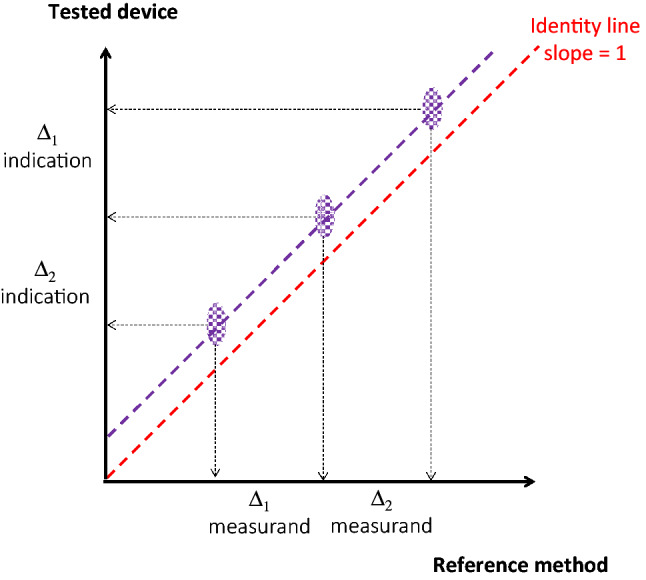
Comparison between the test device and the reference method, based on three sets of paired measurements representing the whole measuring interval of cardiac output (1.5–4.5 L/min). Purple areas show the uncertainty as derived by the reference 2 SEM on the x-axis and the test device 2 SEM on the y-axis. The data of the central point are those shown in Fig. 5. In this example, the precisions of both technologies remain constant over the measuring interval (same purple areas). The test device shows a good sensitivity (Δ_1_ indication/Δ_1_ measurand = Δ_2_ indication/Δ_2_ measurand = 1) with a regression curve (purple dotted line) close to the perfect identity shown by the red dotted line. Therefore, the instrumental bias shown by the test device is constant (offset)

Once these properties have been studied for one central value of the measurand, other values of the measurand must be tested, preferably the minimum and the maximum values of the prescribed measuring interval. For example, if we imagine that the measurand studied in Fig. 6 is cardiac output, other experiments must be done to verify the instrumental precisions and biases for low and high values (for example cardiac output = 1.5 and 4.5 L/min). This allows estimating the sensitivity and linearity of the test device (Fig. 7).

#### Uncertainty

**Fig. 8:**
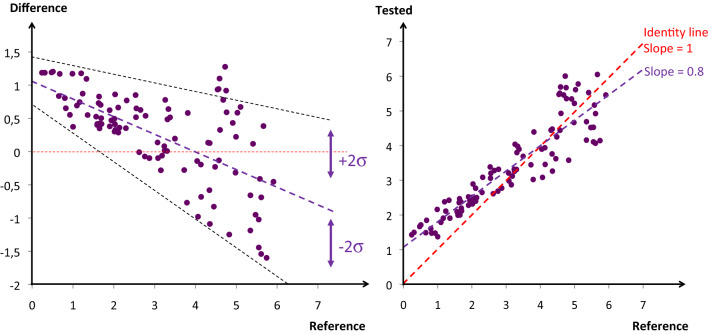
On the left panel a modified Bland & Altman representation is shown. One hundred inter- and intra-patients paired measurements are reported. In this example, indicating the mean bias and the variability of the bias, the instrumental bias of the test device is decreasing from low to high values of the measurand. In addition, the uncertainty (combining imprecision, non-sensitivity, and interpatient variability of the bias) is increasing proportionally to the value of the measurand. On the right panel, same data reported on a regression plot showing the same non-idealities (with different scales). The imperfect sensitivity is shown by a regression slope < 1 and indicates the need for a better gain

Figure 7 shows a set of experiments on the same patient, in the same location, with the same investigator, and the same device. Adding more diversity in each of the three last variables will enlarge the area of imprecision which is referred to as reproducibility of measurements. Adding more than one patient (referred to as the inter-patient variability) represents another source of variability to the device uncertainty. Since it is quite difficult to imagine the validation of a single device, covering the whole measuring interval (at least three sets of measurements) in a given patient, it is impossible to test different investigators, different devices, and different locations (reproducibility) on the same patient. That’s why we are often limited to test a technology using different devices, in different locations, calibrated with different chains, compared to different references, with different investigators, and in different patients. In that suboptimal situation, each piece of diversity adds its own variability and it becomes critical (even inappropriate) to speak of the instrumental bias or the instrumental precision of a technology. It would be preferable to use the general term of uncertainty, with its systematic component and variability, and its random component and variability (Table 1). Moreover, since it is often difficult to manipulate the physiologic variable (measurand) of a patient, the whole measuring interval may be investigated using the inter-patient physiological or pathological variability. Paired measurements of the measurand using a reference method and a device to be tested may be pooled together, coming from different patients, locations, investigators, devices, etc. A regression line between the reference and the test device, or a modified Bland and Altman representation may be used, plotting on the y-axis the difference between the reference and the test devices but only the reference value on the x-axis, since it is supposed to represent the true value (Fig. 8) [4].

**Table 1:**
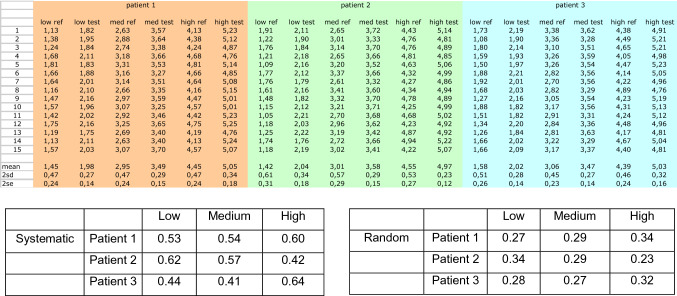
Same comparison than in Fig. 6 (with low, medium and high values of the measuring interval) in three different hypothetical patients where constant random and systemic errors have been computed

All data are shown in the top table. The averaged results are summarized in the tables below. According to the patient and to the level of the measurand, the systemic bias ranges from 0.42 to 0.64 (global mean = 0.53 ± 0.09) and the random error ranges from 0.23 to 0.34 (global mean = 0.29 ± 0.04). This validation procedure, if real, would have shown that any change over the measuring interval of the test device >0.33 is real and not due to chance alone; also that there is a constant offset that must be corrected for

The step response time validation requires a constant quantity of the measurand, followed by a sudden change (preferably instantaneous) at a known start time. In clinical practice, this sudden change may not be easy to provoke and to prove. Natural changes can be used if the test device can be compared with a reference method with a known fast response time. The reference method for validating the step time response of a measuring device may be different than the reference method for measurements. For example, for validating the step response time of a device measuring cardiac output, fast changes in mixed venous blood oxygen saturation (SvO_2_) or invasive blood pressure can be chosen as the reference if other components than SvO_2_ and blood pressure can be stabilized.

#### Stability

Although instrumental drift is easy to evidence, stability may affect all properties of a measuring device and contributes to the uncertainty of measurements. A complete analysis of stability requires restarting the validation process after a certain delay to verify that the same performances are obtained. The specified period of time where the stability must be checked depends on the measurand and on the clinical use of the measuring device, ranging from several hours (blood pressure, SpO_2_, SvO_2_, cardiac output) to months (blood gas analyzers, thermometers, mechanical ventilators, …). This is easier when the measurand is well known (coming from a bench or a calibrated device). When the validation process involves real patients, the inter-patient variability necessarily enlarges the uncertainty and may hide a small systematic drift.

## Practical considerations for validations procedures

Providing specific recommendations how to plan, perform, analyze, and report method comparison studies in perioperative and intensive care medicine is far from being trivial because different variables (measurands) assessed in these settings have distinctly different properties, dynamics, normal ranges etc. Therefore, when analyzing the agreement between a test method and a reference method, specific aspects must be considered depending on the measurand of interest. For example, evaluating the agreement between two methods for the measurement of blood glucose [5] requires different statistical tests than comparing methods for blood pressure [6] or cardiac output [7].

Giving recommendations on how to perform validation procedures and method comparison studies for various different variables used in perioperative and intensive care medicine is beyond the scope of this article. Nevertheless, we want to emphasize that the metrological terms and definitions described in the article are not only theoretical concepts but concepts that are essential when performing and reporting validation studies. For instance, cardiac output is a key variable in the treatment of high-risk surgical patients and critically ill patients with circulatory shock, and there are different methods to assess cardiac output in clinical practice [8]. Let’s suppose that we want to evaluate a method (test method) for cardiac output as measurand and describe its measurement quality criteria. The following metrological concepts should be considered and reported in order to adequately report research results in cardiac output validation studies.

### Instrumental precision of test method (random error)

- On a unique patient in a steady state, collect as much as possible repeated indications using the test method during a short period of time. Since during this short period of time, the true cardiac output is supposed to be relatively constant, the 2σ and 2σ/µ of the test method indications estimate the random error of measurement (repeatability). However, even in clinically stable patients, the true value of any hemodynamic variable is never completely constant over several cardiac and respiratory cycles. Therefore, from a strict statistical point of view, we have to deal with repeated measurements of multiple (changing) true values and not with repeated measurements of a single (constant) true value [3].
- Then, restart the same process with different patients for deriving the inter-patient random error as mean 2σ’ and mean 2σ’/µ’ (intermediate precision).
- Then, in theory, one may restart with different operators and different devices for deriving the inter-operator, and inter-device random error as mean 2σ’’ and mean 2σ’’/µ’’ (reproducibility).

### Instrumental bias of test method (systematic error)

- Choose a reference method with a 2SEM_ref_ ideally < 2σ/4. For example, if the test method precision obtained in step 1 is 2σ/µ = 16%, the reference method should reach at least a SEM_ref_ of 4%. Hence, if a continuous thermodilution method with 2σ/µ_ref_ = 20% is chosen as reference method for measuring cardiac output, then 25 measurements (indications) should be obtained (since 20/$\sqrt{25}$ = 4). If a bolus thermodilution method with 2σ/µ_ref_ = 10% is chosen as reference method, then 6 measurements should be obtained (since 10/$\sqrt{6}$ = 4). If a steady state cannot be obtained during the period of time necessary to collect the appropriate number indications, another reference method must be found.
- Compare the test method indications and the reference method indications for at least one low, one medium, and one high value of cardiac output. Then derive the slope and compare it with the identity line (sensibility and linearity).
- Repeat this procedure in a series of different patients to derive the mean bias, mean sensitivity, and mean linearity and their variabilities.

### Step response time

- Choose a reference method with a fast step response time for measuring cardiac output (not necessarily the same method used for point 2). Indeed, the reference method is not only specific of the measurand but also of the quality criteria. Therefore, any hemodynamic variable closely linked to cardiac output with a very good step response time can be used to study the time delay of a cardiac output indication, if physiologically linked, for example invasive arterial blood pressure. Of course, during the short time of the experiment, other components of the blood pressure must be constant.
- Choose as start time the sudden change in blood pressure following a sudden hemodynamic intervention (for example a lung recruitment test with high level of PEEP). This change is also indicative of the cardiac output change.
- Measure the time delay between the change in blood pressure and the change of the test method indication.
- Repeat this in a series of different patients to derive the mean time and variability.

### Stability of precision, trueness, and step response time

- Restart 2), 3) and 4) during a specified period of time to evidence an eventual drift in the quality criteria mentioned above.

## Conclusions

In perioperative and intensive care medicine, a specific independent evaluation of metrological properties is not actually mandatory as of yet. One of the reasons may be the lack of consensus among physicians, scientists, and scientific societies on which are the recommended qualification procedures. Recently, the European Union published a new directive on medical devices [9], focusing mainly on safety. However, it is mentioned that in the case of devices with a measuring function, the notified body is involved in all aspects relating to the conformity of the device with the metrological requirements [9]. In addition, the rules on clinical investigations should be in line with well-established international guidance in this field such as the International Organization of Standardization (ISO) or Good Clinical Practice (GCP). In line with these recommendations, the international scientific societies of other medical specialties have established metrologic standards. International societies of perioperative and intensive care medicine should start the same process.

## Abbreviations

σ: Standard deviation
SEM: Standard error of the mean
SpO_2_: Arterial oxygen saturation (obtained by pulse oximetry)
SvO_2_: Mixed venous oxygen saturation
FiO_2_: Fraction of inspired oxygen
ISO: International Organization of Standardization
